# Normalizing Large Scale Sensor-Based MWD Data: An Automated Method toward A Unified Database

**DOI:** 10.3390/s24041209

**Published:** 2024-02-14

**Authors:** Abbas Abbaszadeh Shahri, Chunling Shan, Stefan Larsson, Fredrik Johansson

**Affiliations:** 1 Johan Lundberg AB, 754 50 Uppsala, Sweden; 2 Division of Rock Engineering, Tyrens, 118 86 Stockholm, Sweden; chunling.shan@tyrens.se; 3 Division of Soil and Rock Mechanics, Royal Institute of Technology, KTH, 114 28 Stockholm, Sweden; stefan.larsson@byv.kth.se (S.L.); fredrik.johansson@byv.kth.se (F.J.)

**Keywords:** sensor-based data, measurement while drilling (MWD), normalizing index, filtering process, tunneling, Sweden

## Abstract

In the context of geo-infrastructures and specifically tunneling projects, analyzing the large-scale sensor-based measurement-while-drilling (MWD) data plays a pivotal role in assessing rock engineering conditions. However, handling the big MWD data due to multiform stacking is a time-consuming and challenging task. Extracting valuable insights and improving the accuracy of geoengineering interpretations from MWD data necessitates a combination of domain expertise and data science skills in an iterative process. To address these challenges and efficiently normalize and filter out noisy data, an automated processing approach integrating the stepwise technique, mode, and percentile gate bands for both single and peer group-based holes was developed. Subsequently, the mathematical concept of a novel normalizing index for classifying such big datasets was also presented. The visualized results from different geo-infrastructure datasets in Sweden indicated that outliers and noisy data can more efficiently be eliminated using single hole-based normalizing. Additionally, a relational unified PostgreSQL database was created to store and automatically transfer the processed and raw MWD as well as real time grouting data that offers a cost effective and efficient data extraction tool. The generated database is expected to facilitate in-depth investigations and enable application of the artificial intelligence (AI) techniques to predict rock quality conditions and design appropriate support systems based on MWD data.

## 1. Introduction

Measurement while drilling (MWD) is a sensor-based monitoring technology [1]. However, as referenced by [2], the use of MWD as a drill monitoring technique in different geoengineering applications has been well recognized since the 1970s. Real-time drilling data captured by MWD can provide detailed design insights for geologic formations through processing and interpretation. [3]. Depending on the type of drilling rig, several parameters, i.e., thrust, air pressure, feed pressure, percussion pressure, rotation speed, penetration rate, torque, flushing pressure, flushing flow, drilling depth, and time are measured [4]. The immediacy and relative cheapness of data acquisition using the embedded different sensors in the drilling rig is the main attractiveness of this technology [5].

**Figure 1:**
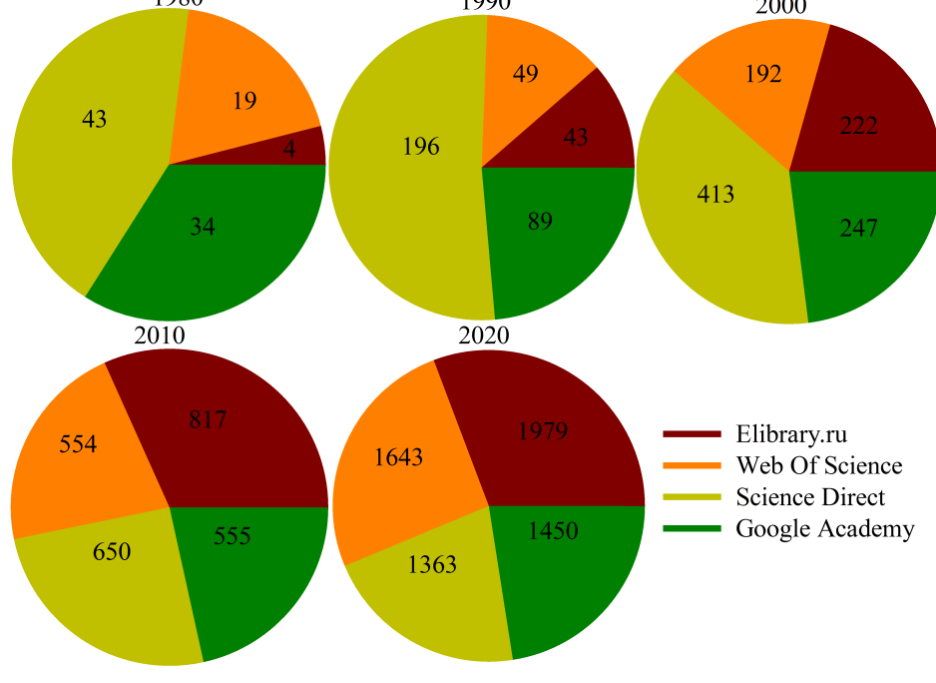
Increasing trend of using MWD data in geoengineering application in last five decades (after [14]).

Currently, organization and interpretation of the collected MWD data have successfully been applied on geo-infrastructures in several countries like Sweden [4,6], USA [7], Norway [8,9], Spain [10], Canada [11,12], and Russia [13]. Figure 1 shows the increased cumulative trend of the geoengineering application of MWD data in recent years.

Technically, standardization of data formats [15], data integration [16], data cleansing [17], metadata management [18], cloud-based solutions [19], and application programming interfaces (APIs) [20] are the most commonly used approaches for processing and managing a centralized MWD database in geoengineering. Overall, these methods aim to define a consistent form of MWD data processing that can be integrated and shared across different systems and platforms. However, in terms of data analytical systems, the MWD data is a typical representation of complex large-scale and big data in geoengineering applications that cannot easily be stored in traditional databases. Accordingly, the outliers of such metadata require appropriate removal (filtering) and scaling (normalizing) for consistent interpretation and a further centralized storing location (unified database) to assist quick retrieval of relevant data for analysis. The drilling rig is composed of various tools that interact in complex ways, such as the drill string, bit, and subsurface. This interaction may introduce noise or anomalies in the MWD parameters, which may lead to outliers. Subsequently, the MWD data is typically acquired by embedded sensors near the drill bit, and thereby, the presence of noisy records due to various factors, like the drilling environment/ condition, tool wear, and signal interference cannot be neglected.

The concept of a normalizing process in combination with different calculation methods has been used for solving a variety of decision-making problems in civil engineering [21,22,23]. Table 1 shows the most commonly used normalizing methods including linear transformation [24,25], nonlinear transformation [26,27], vector normalization [28], and logarithmic approach [29]. However, the first analysis of the impact of the applied normalizing method on the results was highlighted by [30] and then [27].

Consequently, establishing a unified MWD database provides a crucial structured tool/framework that ensures data integrity and minimizes redundancy. The unified database also can improve data management, i.e., a centralized location with accessible shared space via regulatory compliance requirements during both operational and research stages. This implies that unification facilitates in-depth physically meaningful interpretation of the retrieved information. These characteristics then provide a consistent and cost-effective data analysis platform for auditing and optimization across data mining and artificial intelligence (AI) approaches to obtain more detailed information on subsurface conditions. As a result, the unified database facilitates collaborations between geoengineers and stakeholders for better communication and promoting more efficient workflows [31,32,33]. Such analysis will then greatly help the geoengineers to identify patterns, and trends and anomalies that allow error elimination to be more based on informed decision-making and operational improvement [14].

Due to a lack of acknowledged capability of big data analysis in geo-modelling problems [34], geoengineers may face obstacles at the initial stage of analysis. This is primarily because they have been neither aware nor equipped to address the encountered challenges. On the other hand, the large amount of geo-data generated during the projects often are annotated manually for the project purposes where the acquired data neither are normalized in the same scale nor filtered properly for outlier removal. Therefore, creating an integrated unified meta-database using an advanced automated procedure covering the filtering and normalizing processes is highly desirable in geoengineering applications. In the current paper, to rescue both high and low bands of MWD data, a novel automated normalizing approach for analyzing the single/peer group-based holes using the mode and average gated bands supplemented by the percentile filtering and different variants of component combinations is presented. With this strategy, a normalized index was also introduced to categorize the accuracy and acceptable performance of the process for each of the recorded MWD components. Practically, the capacity of the suggested procedure was examined on acquired MWD data from two different geo-infrastructure tunnels in Sweden. The results showed that the single hole-based strategy could provide more concise results in outlier removal.

## 2. Material and Methods

### 2.1. Data Source Description

In the current paper, the MWD data from part of two different geo-infrastructure projects in Sweden, namely as väslänken and Stockholm Bypass, titled FSE410, were analyzed. The used datasets from FSE410 also included the real-time grouting supplemented by protocols, i.e., drilling plans and water flow measurements. These supplementary datasets could potentially be the subject of the development of modern AI-based modeling approaches for detailed analysis of the MWD parameters and grouting design.

**Figure 2:**
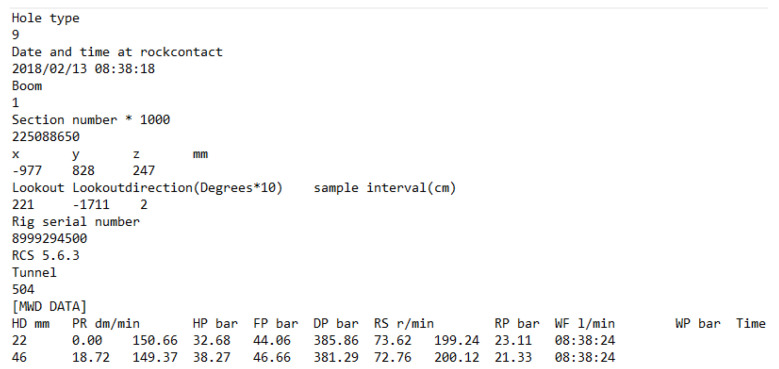
A sample of the format of the raw records of MWD data.

The employed MWD datasets and their units followed a matrix and txt format (Figure 2). The columns show the measured parameters including hole depth (HD, mm), penetration rate (PR, dm/min), percussive pressure (HP, bar), feed pressure (FP, bar), damping pressure (DP, bar), rotation speed (RS, r/min), rotation pressure (RP, bar), water flow (WF, l/min) and water pressure (WP, bar), and the time of operation (hh:mm:ss); the rows present the corresponding measured values of each recorded interval.

### 2.2. Applied Methodology

**Figure 3:**
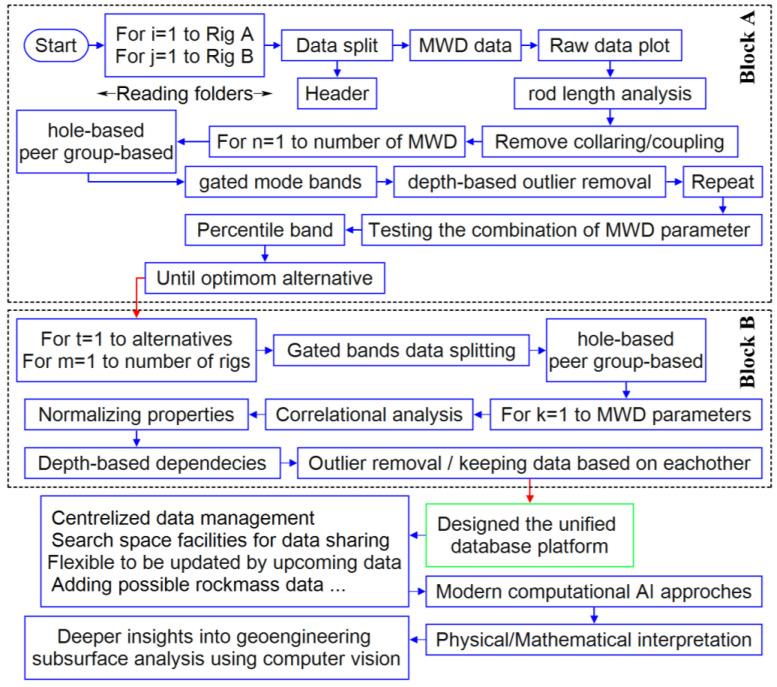
Simplified diagram of the applied automated MWD processing procedure and generating unified database.

The flow diagram of the proposed approach is presented in Figure 3 and entirely coded in Python. Block ‘A’ shows the process of the adopted multi-filtering procedure while Block ‘B’ expresses the implemented framework in the normalizing step. The procedure, due to the presence of several inner nested loops, mimics an automated process, where the input MWD data after selecting the adaptive dynamic filtering and subsequent normalization are transferred to the centralized space to be stored and create the unified database.

This process was designed in such a way that covers both single hole and peer group-based analyses. In the hole-based procedure, each individual MWD data (single hole) is fed, while the peer group is referred to a set of MWD records based on analytically relevant criteria, i.e., the diameter and hole depth that are related to the rod length.

#### 2.2.1. Filtering Procedure in Block A

**Figure 4:**
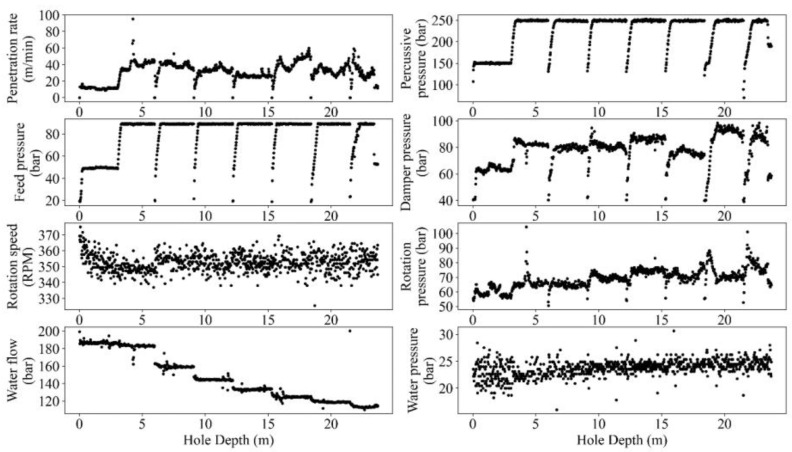
A sample plot of raw MWD records based on rod length in different drilling sequences.

As seen in Figure 4, the analyzed raw data showed different rod lengths in drilling sequences and thus the recommended 0.5 m removal from both sides of the rod by [4] could not be employed. Therefore, as a peer group criterion, the drill rod length should be dealt with as a variable during the filtration process. To solve this issue and obtain an appropriate data split, as presented in Appendix A, a dynamic multi-gated band filtering procedure based on the mode and long-term average statistics was proposed to identify the most appropriate combination of MWD parameters, i.e., PR, HP, DP, HP–FP, DP–HP, HP–DP–FP, etc. The designed bands were then supplemented by a percentile filter.

**Figure 5:**
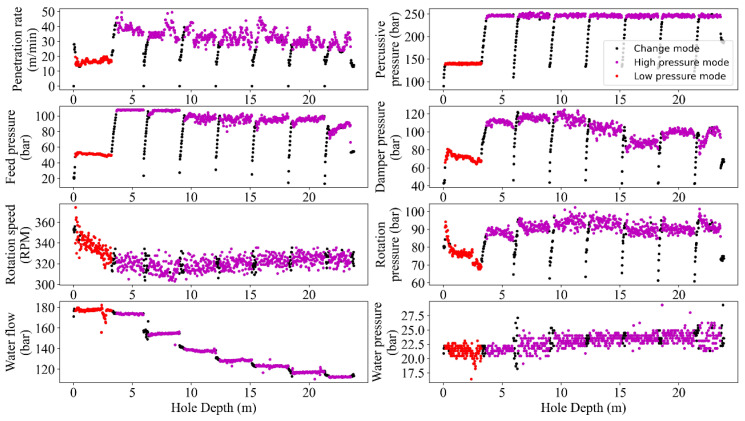
A graphical sample of the carried-out efforts for single hole-based data filtering.

**Figure 6:**
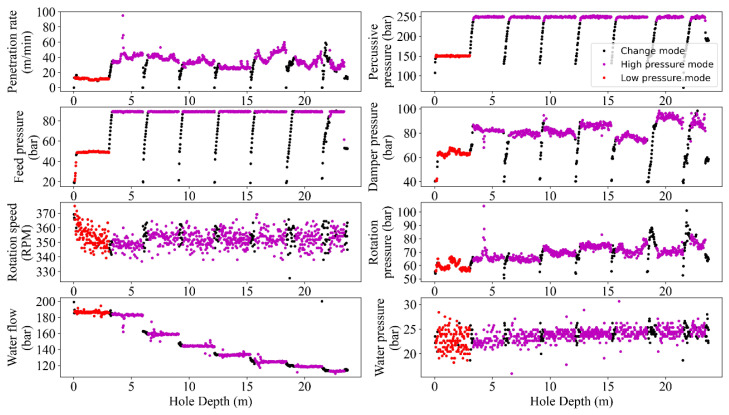
Visualized results of the filtering procedure based on gated bands and modes of the MWD data in accordance to HP.

In the current project, the combination of HP–PR showed the optimum results and thus was selected to define the gated band and percentile filters to remove noises or outliers. This process simultaneously was applied to all the MWD parameters, i.e., removing one data from HP meant eliminating the entire row of data. As seen in Figure 5 and Figure 6, the gated-band-using mode and long-term average supplemented by the percentile showed three states in the datasets, i.e., high/change/low pressure modes. The high-mode data was delineated using a gated band through a combination of mode and average to cover the max of HP, i.e., an interval around the max HP value from peer group drilled holes. Low-pressure data was characterized using the gated band of the mode interval. The rest of the data within the upper/lower gated bands were then attributed as ‘Change mode’, i.e., noisy operational data that due to dependency on the drilling rod length should be excluded in further analysis.

**Figure 7:**
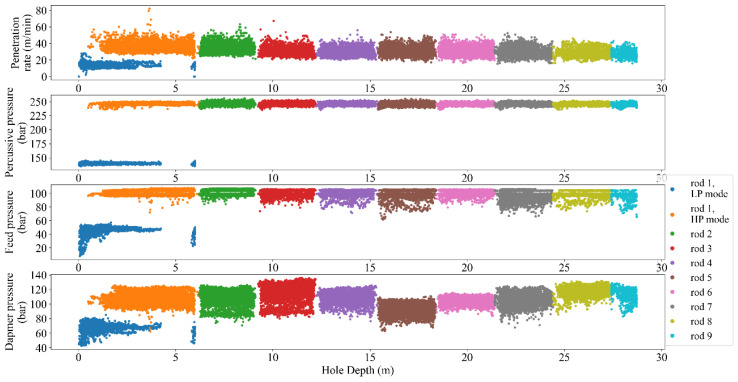
Rod-length checking through splitting of the merged data (checking the mode capability in splitting the high- and low-pressure values for rod 1).

As a result of peer group analysis, a visualized filtering result from one fan in terms of rod length is presented in Figure 7, i.e., the split data from ’rod 1′ into high/low pressure modes for the depth interval of 0–6 m.

#### 2.2.2. Normalizing Procedure in Block B

**Figure 8:**
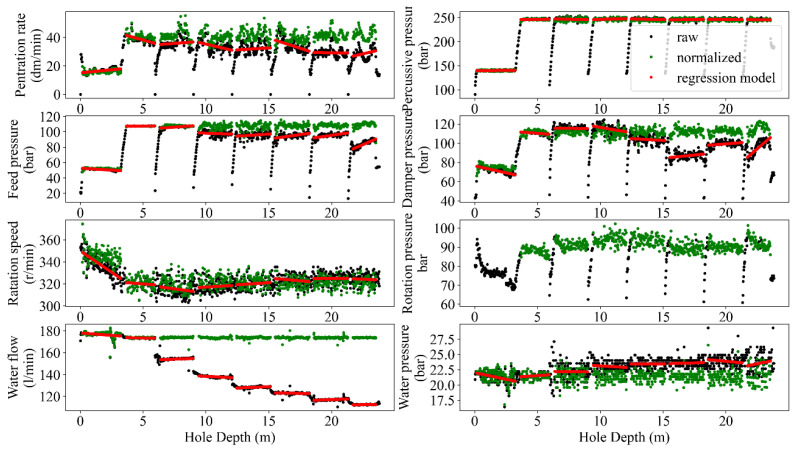
Pattern identification and trend analysis between the normalized and un-normalized MWD data (hole-based).

**Figure 9:**
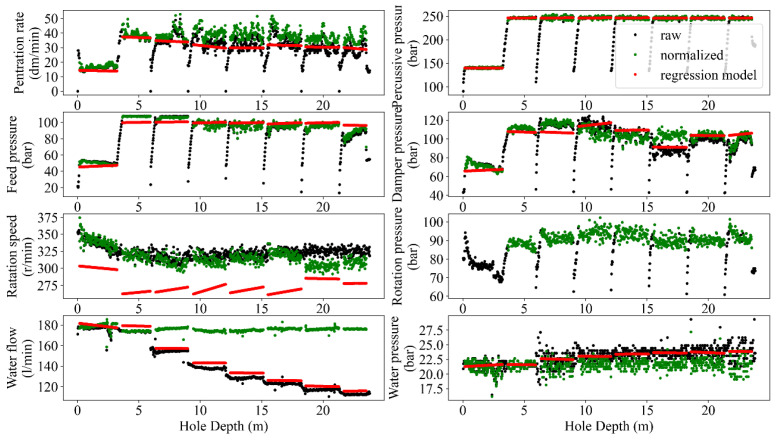
A visualized sample of pattern identification–trend analysis between the normalized and un-normalized MWD data (peer group-based).

**Figure 10:**
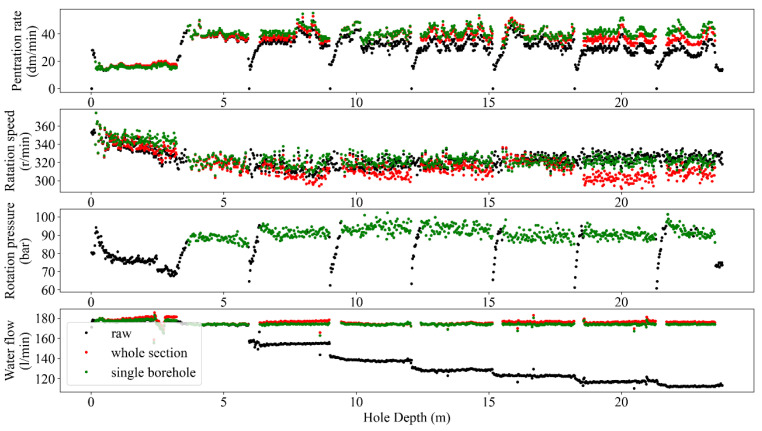
Comparison of two normalization methods for hole depth dependency removal.

The normalization process in MWD data aims to adjust and scale the data to a consistent reference or baseline. This process is commonly used to remove variations in the data caused by differences in rig type, drilling conditions, and other factors, allowing for more accurate analysis and interpretation of the data. Accuracy improvement, providing comparable conditions, sensitivity analysis, and more visualized insights are some of the potential benefits of normalizing MWD data (e.g., [4,10,35]). The result of depth-based normalization for single and peer group holes in terms of raw records (black dots), normalized data after removing the hole depth dependency (green dots), and adopted regressions of each MWD parameter for each rod length (red lines) are presented in Figure 8 and Figure 9. Subsequently, the comparison of the captured results for both single and peer group hole analysis is reflected in Figure 10.

Like any measurement system, MWD tools are not perfect and may have inherent measurement errors. Embedded sensors near the drill bit typically acquire MWD data, but records can be noisy due to various factors like drilling environment, tool wear, and signal interference. These factors can introduce random fluctuations and artifacts into the data and make it challenging to extract accurate and reliable information from the MWD data. From this point of view, filtering the MWD data is a critical task in extracting valuable insights from noisy data to enhance the accuracy of the results analyses, i.e., the improved signal-to-noise ratio and the higher resolution perspective to detect and interpret trends and patterns in the data. Therefore, identifying and handling outliers in MWD data is crucial for maintaining the accuracy of drilling operations and making informed decisions. Mathematically, the MWD data can be filtered using different techniques such as bandpass [36], moving average [37], Kalman [38], and wavelet [39]. However, the choice of filtering technique has a close dependency on the specific application and the characteristics of the MWD data being analyzed [40]. To avoid manual annotating and ensure sustaining the important data during the process, the recommended guidelines by [10] in terms of different combinations of MWD parameters were followed and programmed via automated nested loops to capture the optimum alternatives. Referring to this process, the executed filtering and normalizing showed a degree of improvement in outlier removal caused by rod length, tool geometries, and drilling conditions (Figure 5, Figure 6, Figure 7, Figure 8 and Figure 9).

Normalizing is the process of adjusting or scaling datasets to a standard reference condition to eliminate the effects of variations in drilling circumstances, measurement equipment, and other factors that can affect the data. Since the MWD parameters have different units of measurement, then the normalization aims to obtain comparable scales of criteria values. The MWD data can be normalized using different methods via various parameters like depth normalizing [4,10], time normalizing [41,42,43], lithology normalizing [35], mud weight normalizing [44], tool normalizing [45,46], environmental normalizing [4,14,47], and statistical normalizing [48,49].

### 2.3. Generating A Unified Database

**Figure 11:**
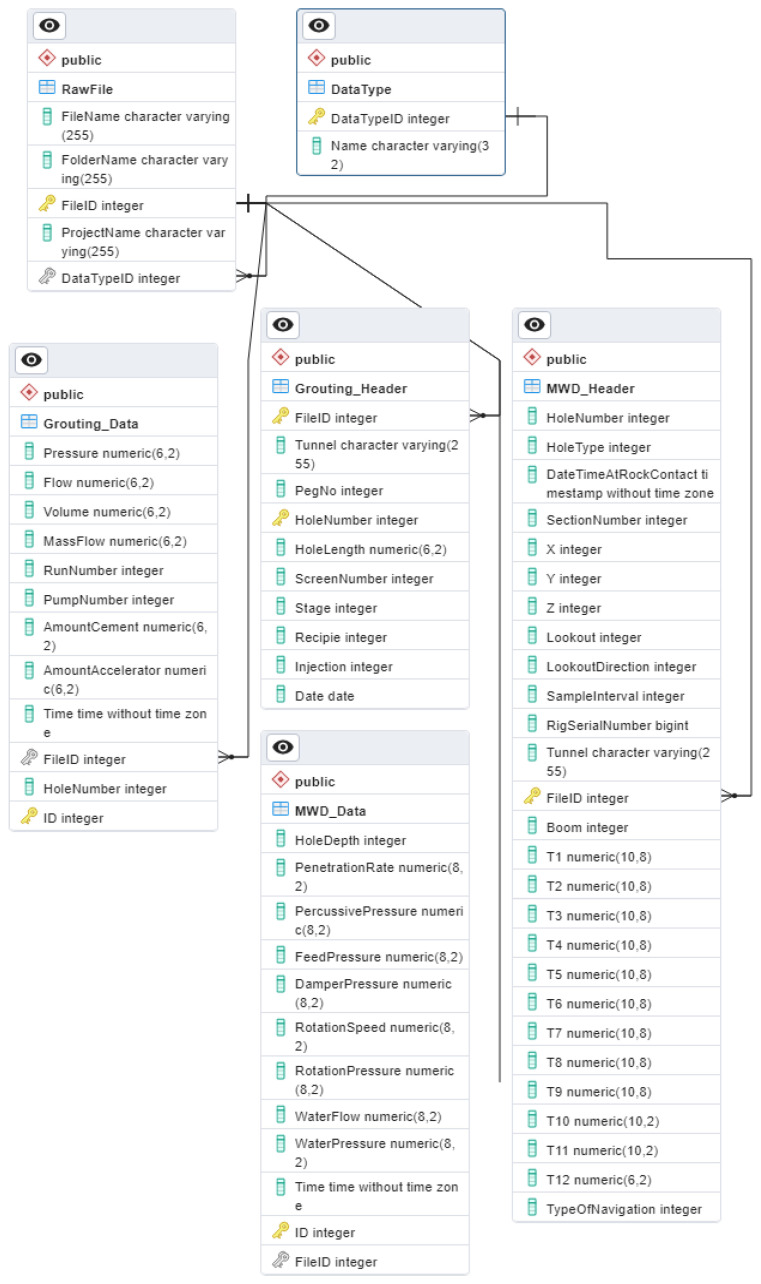
Overview of the designed unified datacenter.

A centralized data center was designed in this study as an accessible place to store normalized and filtered results. The process was performed through the PostreSQL platform because of its robustness and open source object–relational database system. The overview of the designed interface of the datacenter is presented in Figure 11, involving 6 related tables and the connections based on the settings of primary and foreign keys. The ‘
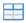
’ corresponds to the table name. The ‘ID’ is the identifier index linked to the original ‘Raw File’. For example, the ID in ‘Data Type’ shows the type of data, i.e., ‘MWD’ or ‘Grouting’ which can be selected in ‘Column Name’. The table of ‘Raw File’ dedicates the information on the name, folder, project, and type of the original uploaded files using ‘File ID’, ‘File Name’, ‘Folder Name’, ‘Project Name’, and ‘Data Type ID’. The tables of ‘MWD_header’ and ‘Grouting_header’ store the information of the header of each data type that is linked to the corresponding file in the table of ‘Raw File’ via ‘File ID’. Accordingly, columns T1–T9 are the three-dimensional rotation matrices of the drill wreath for controlling the spatial direction, and columns T10–T12 denote the absolute coordinates of the starting point of the borehole. The (‘
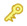
’) shows the unique identity of each row in that table while (‘
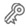
’) represents a set of attributes in a table that refers to the (‘
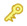
’) of another table. These two keys connect the 6 tables together and enable users to extract data efficiently from different tables at the same time. Such utilities provide efficient choices to extract both MWD and grouting data through different query conditions and specific field ID values.

This database, due to the developed automated coding, can continuously be updated using new upcoming data which significantly can facilitate in-depth investigations using modern computational approaches like AI. The designed database currently includes two types of data, the MWD (7252 file, 7252 boreholes, 60,110,094 data) and real-time grouting (1583 file, 39,766 boreholes, 6,814,391 data). This database currently is located in the Tyréns computer center and can easily be linked to other servers or cloud platforms.

## 3. Discussion

Despite the success of the filtering and normalizing procedure, some of the outliers, i.e., deviated data from the trend of the MWD records, still remained (Figure 5). Technically, during the drilling sequences degradation of wear may influence the sensors accuracies leading to outlier records [50]. On the other hand, formation heterogeneity and subsurface variability, i.e., changes in rock formations and the presence of fractures, can result in unexpected records and thus outliers in the MWD parameters [51]. Furthermore, the complex interactions between components and employed tools in the drilling rigs (drill string, bit, and the subsurface) can introduce noise or anomalies in the MWD parameters, leading to outliers [52]. The accuracy of interpreting MWD records is affected by the depth of drilling. The deeper the depth, the greater the hydrostatic pressure; this can impact the performance of downhole sensors. This, in turn, may affect the accuracy of MWD records, resulting in outlier records [45,46]. Moreover, the problem of vibration and shock also should be considered, because the deeper the drilling, the more challenging conditions, i.e., harder rocks. Therefore, increased vibration and shock loads on the drilling tools can influence the reliability of sensors leading to outliers [53]. Subsequently, real-time data transmission from downhole sensors due to signal interference can corrupt the data, resulting in outliers, where the longer the drill strings, the more signal attenuation and data transmission delays, or potential signal loss in the received data [54]. The influence of operational worker errors in data acquisition and recording also is another potential source of recorded outliers [4].

Following Figure 3, the adopted regressions of each MWD parameter based on the peer group data (Figure 8, Figure 9 and Figure 10) for all the rods concerning identified modes (Figure 6) were conducted. Referring to Figure 8 and Figure 9, both hole and peer group-based results showed the stepwise problem (energy losses in the couplings for the rod extension) in FP and DP at a depth ≥15 m, where the hole-based normalizing could provide more effective stepwise removal than the peer group analysis. However, the low correlation of RP (Figure 9) prevented appropriate depth-normalizing, and thereby, the stepwise problem for a depth ≥15 m was not treated like FP and DP. An overview of the compared methods, i.e., hole/peer group-based depth-normalization is shown in Figure 10, which indicates the improper stepwise removal through peer group analysis in RP around 20 m. Such heterogeneity mechanically can be assigned to the drilled rock mass characteristics which induced uncertainties in the records where the peer group considered all of the holes instead of single data in the hole-based approach.

According to the categorized data state conditions (high/low/change mode) based on the combined PR–HP, the mathematical efficiency of the proposed process in noise removal from the recorded data, i.e., improving the signal-to-noise ratio, was approved. However, referring to [10], some of the data that fell within the identified states may have consisted of information on the poor quality rock that was needed for further investigation using other combined parameters. As an example, the combination of RS, WP, and WF may show variations in the rock mass [10]. The relevance of the normalized MWD parameters integrated with other geotechnical information, i.e., rock mass characteristics and geological mapping, can be evaluated using the sensitivity analysis to pursue how changes might be reflected in the MWD data. Therefore, deeper analysis of normalized MWD data can reveal more insights into the anomalies and trends in the formation that may be of interest for drilling (e.g., changes in lithology, porosity, or permeability). This is an important key for geoengineers because it provides a tool to compare MWD data across different holes/depths and rigs, allowing for a better understanding of the physical properties of the formation being drilled. Overall, physically meaningful interpretation of the normalized MWD data requires an analytical understanding of the executed process (e.g., reference values, applied scaling factors) to identify any biases or errors that may have been introduced during the normalization process to ensure that the data is being analyzed correctly.

Referring to Figure 11, the embedded possibilities dedicate a time/cost-effective tool for big data management for more detailed operational and research analyses through a centralized location that can continuously be updated using new data. The presented method as a new technical guideline in geoengineering applications can specify the search strategy in the big data analysis and retrieval protocols. The database considers the implications of the research findings for practice and will help with consensus decisions on areas where evidence is not found. Accordingly, proper integration of such a unified database with geomechanical data can be the backbone of future deeper analyses through advanced computationally intelligent techniques [55]. Consequently, such databases offer more than just insights into the drilling; they also play a crucial role in optimizing the geoengineering operations and performance improvements via a reliable platform in terms of high-resolution 3D subsurface computer vision models based on the rock mass characteristics and geological mapping. However, the limitations of this study can be dealt with in two different aspects. In terms of geoengineering, the site/rock conditions in comparing the MWD data were not analyzed and will be carried out in future work. From the computer point of view, the problems associated with data redundancy, data inconsistency, and attributes for accessing files were handled but by the expansion of the created data center, concerns like database failure, hardware, and upgrading cost should also be considered.

## 4. Conclusions

In the current project, an entire automated process for filtering, normalizing, and database creation for big MWD data in both hole and peer group-based was developed and presented. A combination of PR–HP parameters was identified as the optimum choice for the filtering procedure. The distinguished states in data (high/low/change mode) using the adopted mode, long-term average and percentile-gated bands showed an efficient role in the removal of the noisy data caused by rig components, i.e., collaring and coupling effects from rod extensions. The applicability of the normalizing process in removing the hole depth dependencies of MWD data was evaluated using different correlational analyses based on the rod length. As a result, the hole-based normalizing method showed better performance in removing the depth dependencies and stepwise problem in the MWD data. However, data splitting for each rod with different length enabled the peer group analysis for more efficient filtering/normalizing of the MWD data. The presented procedure could generally be applied to any retrieved MWD data from each drill rig. The established MWD data center could structure and manage a large amount of MWD and grouting data to facilitate storing and extracting both MWD and grouting data. The generated datacenter mimics the big data characteristic (volume, value, variety, velocity, and veracity), which can not only be continuously updated by upcoming data but also the users via the designed queries are able to extract the desired data. It is of great importance for reference tools for further deeper analyses through modern approaches, i.e., AI modeling, that incorporation with other geomechanical data sources can provide more accurate and realistic physical interpretation from MWD and grouting data.

## Figures and Tables

**Table 1:** The common normalizing methods.

Normalizing Method	Preferred Interval	Note
Vector [28]	$\left[1-\frac{a_{ij}}{\sqrt{\sum_{i=1}^{m}a_{ij}^{2}}},\;\frac{a_{ij}}{\sqrt{\sum_{i=1}^{m}a_{ij}^{2}}}\;\right]$	The ratio of values remains constant within interval [0, 1]
Linear [25]	$\left[\frac{{max\;a}_{ij-}a_{ij}}{{max\;a}_{ij-}min{\;a}_{ij}},\;\frac{a_{ij}-min{\;a}_{ij}}{{max\;a}_{ij-}min\;a_{ij}}\;\right]$	The calculated values are dependent on the size of interval [maxa_ij_, mina_ij_]
[24]	$\left[1-\left|\frac{min\;a_{ij}-a_{ij}}{{max\;a}_{ij-}min{\;a}_{ij}}\right|,\;1-\left|\frac{max{\;a}_{ij}-a_{ij}}{max\;a_{ij}}\right|\;\right]$	Limited to interval [0, 1]
Nonlinear [26]	$\left[{\left(\frac{{min\;a}_{ij}}{a_{ij}}\right)}^{3},\;{\left(\frac{a_{ij}}{{max\;a}_{ij}}\right)}^{2}\right]$	The values are diminished more than when using other methods
Logarithmic [29]	$\left[\frac{1-\frac{ln(a_{ij})}{ln\left(\prod_{i=1}^{n}a_{ij}\right)}}{n-1},\frac{ln(a_{ij})}{ln\left(\prod_{i=1}^{n}a_{ij}\right)}\;\right]$	The sum of normalized criterion values is always 1

**Figure A1:**
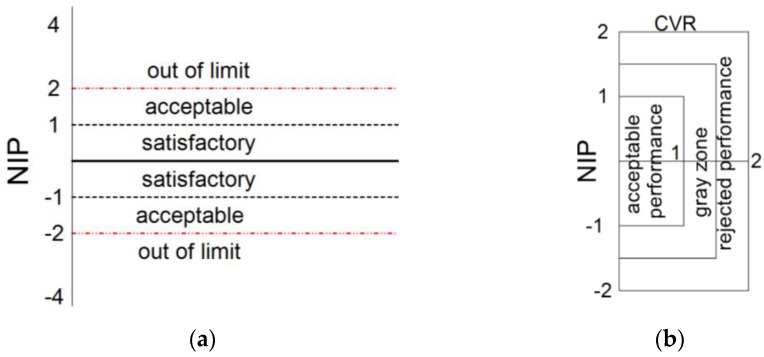
Graphical plot and schemes provided to follow (**a**) Description data removal using *NIP* limits and (**b**) area analysis of the used data based on CVR for outlier elimination.

## Data Availability

Data are stored in Tyréns computer center, Stockholm, Sweden, and will not be shared.

## Appendix A

The expected value of any discrete variable (E(xi)) in each MWD record mathematically is a real number and can be obtained by:

(A1) $$(E\left(x_{i}\right))=\sum_{n}x_{i}P\left(x_{i}\right)\to where;\sum_{i}P\left(x_{i}\right)=1\;$$

where, *P(xi)* is the probability of each discrete parameter *x_i_* in *n* records at any MWD data. On the other hand, *E(xi)* mathematically shows the long-term average or mean (symbolized as μ), which would be expected over the long term of MWD records. Referring to the law of large numbers, the mean ($\bar{x}$) converges to the *E(x)* and thus to the average of the whole records as the number of repetitions approaches infinity [56,57]. Thereby, $\bar{x}$ and variance ($\sigma_{x_{i}}^{2}$) can be calculated using average or a weighted average data as:

(A2) $$\bar{x}=\sum_{i=1}^{N}\frac{x_{i}}{N}=\sum \left(x_{i}\times \frac{n_{i}}{N}\right)\to \bar{x}=\sum \left(x_{i}\times {weight}_{i}\right)$$

(A3) $$\sigma_{x_{i}}^{2}=\sum_{i=1}^{N}\frac{{\left(x_{i}-\bar{x}\right)}^{2}}{N}=\sum_{i=1}^{N}\left[\frac{n_{i}}{N}\times {\left(x_{i}-\bar{x}\right)}^{2}\right]=\sum_{i=1}^{N}\left[{\left(x_{i}-\bar{x}\right)}^{2}\times {weight}_{i}\right]$$

where, *x_i_*: value of observation *i*, *n_i_* = number of observations with value *x_i_*, *N* = total number of observations, $\bar{x}$: population mean, and *n_i_*/*N* is the weight.

Based on the Equations (A2) and (A3), the weights show the number of records among the population and this concept mathematically can be interpreted as mode statistic because it refers to the number that appears the most in a dataset, where depending on the distribution, a set of MWD data may have one/more than one/no mode. On the other hand, the median as the middle number of a given MWD dataset is much more effective than a mean because it eliminates the outliers through the 3 (median) = mode + 2 (mean). Due to the large number of recorded MWD data, there was more than a single mean and variance in the data. Consequently, developing and extending the normalizing procedure to more than a single mean and variance then allowed for detecting the modes of data for jointly normalizing samples that share common features. Therefore, the procedure was carried out based on assigning the gating bands in any mini batch and then normalizing each sample with estimators for the corresponding ranges of modes in both the upper and lower bands. Mathematically, such an approach can cover the mean and median of data in each component of MWD.

Therefore, logically the term $x_{i}-\mu_{i}$ is a normalizing factor that can characterize the ‘Rig effect’.

Each single MWD data has its own individual mean and variance (Equations (A2) and (A3)). Accordingly, due to considering the *E(x)*, Bernoulli probability, and thus binomial distribution, the factor *μ_i_* statistically represents a combination of mean, mode and median of each individual component. Referring to the relation of these statistics in binomial distribution [58], due to the difference of median and mode, the mean lies in between. Therefore, for each individual record of MWD data, gated bands for both the upper and lower observed modes can be defined to cover the mean of each recorded parameter and sustain the median respectively for more efficient removal of the outliers.

Considering the peer group analysis, the measured error is estimated based on the difference between the MWD result and the value of the peer group mean from each of the drilling rods. Accordingly, the bias is estimated as the root mean squared of the measured error from the surveys in each peer group and expressed as a percentage of the total allowable error. In peer group analysis, the distance of the recorded MWD results from the consensus mean for quantification of the inaccuracy of the recorded MWD data and is defined as follows:

(A4) $$NIP=\frac{x_{i}-\mu_{i}}{S_{i}}$$

where *NIP* denotes the normalized index parameter. $\mu_{i}$ and $S_{i}$ are the mean and standard deviation of the peer group. In the case of each parameter without a peer group then *NIP* can be written as:

(A5) $$NIP=\frac{x_{i}-\mu_{i}}{\sigma_{i}}$$

The control limits of *NIP* are zero ± 2 *NIP*. On the other hand, $\sigma_{i}$ cannot be used to compare the variance of different distributions or distributions with a different mean. Therefore, for comparison reasons the coefficient of variation (*CV*%) is being used.

(A6) $${CV}_{i}=\frac{\sigma_{i}}{\mu_{i}}$$

In the case of peer group, the *CVR* (*CV* ratio) should be considered. Therefore:

(A7) $$CVR=\frac{CV\;of\;recorded\;MWD\;data}{CV\;of\;peer\;group}$$

To remove the outliers, then combination of *CVR* and *NIP* can be presented as follows:

Another aspect in this project was assigned to process assessment and result qualities, where the normalized MWD data could be used to compare data across acquired datasets at different times. To show whether normalization could help to improve the quality of MWD data by removing noise and other unwanted variations, a comparative peer group-based analysis for all the monitored datasets using the introduced Figure 3 and Figure 4 corresponding to Equations (A4)–(A7) were carried out and reflected in Table A1 and Table A2. Referring to achieved results, the presented index in Figure A1a was more sensitive to outliers than Figure A1b. The reason mathematically was assigned to *CV*. This physically also makes sense because using the *CV* for peer group-based analysis considers all rod lengths and corresponding drops. By taking these factors into account, analysts can gain valuable insights into the drilling process and the properties of the formation being drilled. However, it is still important to emphasize assessing the quality of the data to identify any sources of uncertainty or error that may impact the analysis.

**Table A1:** Analyzed MWD data using presented *NIP* and *CVR* plots- Väst länken data.

MWD Parameter	NIP-Väst Länken			NIP-CVR Väst Länken		
	Satisfactory	Acceptable	Out of Limit	Acc. Performance	Gray Zone	Out of Limit
PR dm/min	133,300	8126	2117	141,426	0	10,243
HP bar	136,559	3175	11,935	136,559	0	15,110
FP bar	140,450	3174	8045	140,450	0	11,219
DP bar	104,819	38,844	8006	104,819	0	46,850
Rs r/min	110,447	32,508	8714	110,447	0	41,222
RP bar	103,328	42,012	6329	103,328	0	48,341
WF l/min	129,425	12,672	9572	129,425	0	22,244
WP bar	124,121	17,992	9572	124,121	0	27,548

Number of analyzed data: 151,669 data.

**Table A2:** Analyzed MWD data using presented *NIP* and *CVR* plots- FSE410 data.

MWD Parameter	NIP-FSE410			NIP-CVR FSE410		
	Satisfactory	Acceptable	Out of Limit	Acc. Performance	Gray Zone	Out of Limit
PR dm/min	4,137,640	1,111,975	130,073	4,137,640	0	1,242,048
HP bar	4,337,126	855,113	187,449	4,337,126	0	1,042,562
FP bar	3,703,051	1,494,882	181,755	3,703,051	0	1,866,593
DP bar	3,513,095	1,652,843	213,750	3,513,095	0	1,866,593
Rs r/min	4,642,513	202,275	534,900	4,642,513	0	737,175
RP bar	3,760,962	1,389,361	229,365	3,760,962	0	1,618,726
WF l/min	3,580,175	1,687,855	111,658	3,580,175	0	1,799,513
WP bar	4,494,233	523,568	361,887	4,494,233	0	885,455

Number of analyzed data: 5,379,688 data.
